# Supplementary material for: Promoting vigorous intermittent lifestyle physical activity (vilpa) in middle-aged adults: an evaluation of the movsnax mobile app
Source: BMC Public Health. 2024 Aug 12;24:2182. doi: 10.1186/s12889-024-19549-9 (PMC11318164; doi:10.1186/s12889-024-19549-9)
Supplement: Supplementary file 1 — Supplementary Material 1: Table S1. Part A survey questions [file 12889_2024_19549_MOESM1_ESM.pdf]

Table S1. Part A survey questions

Which of the following functions did you explore (tick options)? Weekly progress, Adjusting daily VILPA goals, Notifications, VILPA self-reporting, Steps tracking, Others
What did you think about the functionality of the app?
What did you think about the aesthetics or design of the app?
How useful do you think the app will be in promoting VILPA in 40-65-year-old adults who are physically inactive?<br>In your response, please comment on specific functionalities of the app.
How relevant do you think the app will be in promoting VILPA in 40-65-year-old adults who are physically inactive?<br>In your response, please comment on specific functionalities of the app.
How beneficial do you think the app will be in promoting VILPA in 40-65-year-old adults who are physically inactive?<br>In your response, please comment on specific functionalities of the app.
Please describe your overall experience of using the app over the past 7 days – Aesthetics
Please describe your overall experience of using the app over the past 7 days – Practicality
Please describe your overall experience of using the app over the past 7 days – Functionality
Please describe your overall experience of using the app over the past 7 days – Usefulness
What did you like the most about the app?
What did you not like about the app?
How do you think the app could be improved?
Are there any other comments you would like to make about the app?
