# Supplementary material for: Promoting vigorous intermittent lifestyle physical activity (vilpa) in middle-aged adults: an evaluation of the movsnax mobile app
Source: BMC Public Health. 2024 Aug 12;24:2182. doi: 10.1186/s12889-024-19549-9 (PMC11318164; doi:10.1186/s12889-024-19549-9)
Supplement: Supplementary file 2 — Supplementary Material 2: Table S2. Part D interview schedule [file 12889_2024_19549_MOESM2_ESM.pdf]

Additional File 2. Table S2. Part D interview schedule

First, can you tell me a bit about your experiences of using mobile apps in general?
And what about apps designed to increase physical activity
Can you tell me about your experiences of using the VILPA app?
(following up on their responses to the uMARS survey): Your score on X [e.g., functionality] was fairly low– can you elaborate on your reason for this score?
In contrast, in the survey, you scored Y [e.g., engagement] quite high – what was your reason for that?
What, if anything, did you like about the app?
What, if anything, didn't you like about the app?
Did you choose to explore some functions and not others? If so, which and why?
If *the participant indicate they didn't use one or more functions much: what were some reasons or barriers to using them?*
Which functions you did not explore at all? If so, which and why?
How helpful do you think the VILPA app would be to increase your levels of VILPA? In what ways? How?
Which parts of the app would be more/less helpful?
How do you think the app could be improved?
Would you be recommending the app to others? Why, why not?
