# Supplementary material for: Promoting vigorous intermittent lifestyle physical activity (vilpa) in middle-aged adults: an evaluation of the movsnax mobile app
Source: BMC Public Health. 2024 Aug 12;24:2182. doi: 10.1186/s12889-024-19549-9 (PMC11318164; doi:10.1186/s12889-024-19549-9)
Supplement: Supplementary file 5 — Supplementary Material 5: Table S5. Part C Socio-demographic and health characteristics of participants (N = 35) [file 12889_2024_19549_MOESM5_ESM.pdf]

Table S5. Part C Socio-demographic and health characteristics of participants (N=35)

	Values n (%)
**Sex**	
Female	28 (80)
Male	7 (20)
**Age (years)**	
40-45	5 (14.29)
46-50	4 (11.43)
51-55	6 (17.14)
56-60	9 (25.71)
60-65	11 (31.43)
**Ethnicity**	
Oceanian*	19 (54.3)
Southeast Asian	1 (2.9)
Northeast Asian	1 (2.9)
Southern and Central Asian	1 (2.9)
Northwest European	6 (17.1)
Southern and Eastern European	2 (5.7)
Other	5 (14.3)
**Subjective socio-economic status**	
1 (lowest)	1 (2.90)
2	0 (0)
3	2 (5.90)
4	1 (2.90)
5	3 (8.60)
6	7 (20)
7	9 (25.70)
8	4 (11.4)
9	1 (2.90)
10 (highest)	2 (5.70)
**Highest level of education**	
Year 11 or below (incl. certificate I/II)	2 (5.70)
Diploma/Advanced Diploma/Vocational training	7 (20)
Bachelor/Masters/Graduate Diploma	22 (62.90)
Doctoral degree/PhD	2 (5.70)
Other	2 (5.70)
**Marital status**	
Never married	3 (8.60)
Married/de facto	27 (77.10)
Separated/divorced	5 (14.30)
**Work status**	
Full-time	12 (34.30)
Part-time	9 (25.70)
Retired	8 (22.90)
Other	6 (17.10)
**Current health status**	
Poor	1 (2.90)

Fair	4 (11.40)
Good	11 (31.40)
Very good	17 (48.60)
Excellent	2 (5.70)
**Health issues**	
Yes	18 (51.40)
No	17 (48.60)
**Body Mass Index**	
<25	14 (41.18)
>25-<30	10 (29.41)
>30	10 (29.41)
**Weight perception**	
Very underweight	0 (0)
Slightly underweight	1 (2.90)
About the right weight	10 (28.60)
Slightly overweight	11 (31.40)
Very overweight	13 (37.10)

*n=18 identified as Australian; n=1 as Aboriginal/Australian South Sea Islander
