# Supplementary material for: Promoting vigorous intermittent lifestyle physical activity (vilpa) in middle-aged adults: an evaluation of the movsnax mobile app
Source: BMC Public Health. 2024 Aug 12;24:2182. doi: 10.1186/s12889-024-19549-9 (PMC11318164; doi:10.1186/s12889-024-19549-9)
Supplement: Supplementary file 6 — Supplementary Material 6: Table S6. Part D Socio-demographic and health characteristics of participants (N = 15) [file 12889_2024_19549_MOESM6_ESM.pdf]

Table S6. Part D Socio-demographic and health characteristics of participants (*N*=15)

	**Values n (%)**
**Sex**	
Female	11 (73.3)
Male	4 (26.7)
**Age (years)**	
40-45	1 (6.7)
46-50	4 (26.7)
51-55	3 (20)
56-60	5 (33.3)
61-65	2 (13.3)
**Ethnicity**	
Oceanian*	8 (53.3)
Southeast Asian (Southeast, Northeast, Southern and Central)	0 (0)
Northwest European	3 (20)
Southern and Eastern European	1 (6.7)
Other	3 (20)
**Subjective socio-economic status+**	
1 (lowest)	0 (0)
2	0 (0)
3	1 (0)
4	1 (6.7)
5	2 (13.3)
6	5 (33.3)
7	3 (20)
8	0 (0)
9	1 (6.7)
10 (highest)	0 (0)
**Highest level of education**	
Year 11 or below (incl. certificate I/II)	0 (0)
Diploma/Advanced Diploma/Vocational training	4 (26.7)
Bachelor/Masters/Graduate Diploma	10 (66.7)
Doctoral degree/PhD	1 (6.7)
Other	0 (0)
**Marital status**	
Never married	0 (0)
Married/de facto	13 (86.7)
Separated/divorced	2 (13.3)
**Work status**	
Full-time	4 (26.7)
Part-time	2 (13.3)
Retired	4 (26.7)
Other	5 (33.3)
**Current health status**	
Poor	0 (0)
Fair	2 (13.3)
Good	5 (33.3)

Very good	8 (53.3)
Excellent	0 (0)
**Health issues**	
Yes	8 (53.3)
No	7 (46.7)
**Body Mass Index**	
<25	7 (46.7)
>25-<30	6 (40)
>30	2 (13.3)
**Weight perception**	
Very underweight	0 (0)
Slightly underweight	0 (0)
About the right weight	6 (40)
Slightly overweight	7 (46.7)
Very overweight	2 (13.3)

*All identified as Australian Caucasian; +*n*=2 participants did not respond to this question
